# Case Report: A Novel Mutation Leading to 11-β Hydroxylase Deficiency in a Female Patient

**DOI:** 10.2174/1871530322666221007145410

**Published:** 2023-04-05

**Authors:** Burak Ozbas, Mikail Demir, Huseyin Dursun, Izem Sahin, Aysa Hacioglu, Zuleyha Karaca, Munis Dundar, Kursad Unluhizarci

**Affiliations:** 1 Department of Endocrinology, Erciyes University Medical School, Kayseri, Turkey;; 2 Department of Medical Genetics, Erciyes University Medical School, Kayseri, Turkey

**Keywords:** Congenital adrenal hyperplasia, 11-beta hydroxylase deficiency, 11-deoxycortysol, CYP11B1 gene, mineralocorticoid pressure levels

## Abstract

**Background:**

11β hydroxylase deficiency (11βOHD) ranks as the second most common enzyme deficiency that causes congenital adrenal hyperplasia. Depending on the severity of the enzyme deficiency, it can lead to cortisol deficiency, androgen excess and hypertension due to increased mineralocorticoid precursor levels. Many different types of mutations in the CYP11B1 gene located on chromosome 8q24.3 have been shown to cause 11βOHD. Here, we report a novel missense mutation that leads to 11βOHD in a female patient.

**Case Presentation:**

A 35-year-old female patient was admitted to the Endocrinology Department with a complaint of abdominal pain. The patient had a history of genital reconstruction surgery twice in childhood. On physical examination, an abdominal mass was detected. Laboratory examination of the patient revealed low levels of cortisol, potassium and high levels of ACTH, 11-deoxycortisol and androstenedione, suggesting 11βOHD. Genotyping showed a novel homozygous missense mutation (c.1385T>C L462P variant) detected on the 8^th^ chromosome where the CYP11B1 gene is located. Glucocorticoid therapy was commenced for the patient whose diagnosis of 11βOHD was confirmed by both hormonal and genetic tests. A mass originating from the left adrenal gland with the largest diameter of 7 cm was compatible with myelolipoma.

**Conclusion:**

In this case report, we aimed to contribute to the literature by reporting a new missense mutation in the CYP11B1 gene, leading to classic type 11βOHD that has not been described before.

## 1 INTRODUCTION

Congenital adrenal hyperplasia (CAH) is a group of diseases in which impaired cortisol synthesis occurs due to the defects in enzymes responsible for adrenal steroidogenesis [1]. Deficiencies in enzymes (21 hydroxylase, 11β-hydroxylase, 17α-hydroxylase, 3β-hydroxysteroid dehydro-genase type 2, steroidogenic acute regulatory protein, P450 cholesterol side-chain cleavage enzyme and P450 oxidoreductase) necessary for steroid biosynthesis lead to CAH, which is one of the most common genetic diseases [2]. These autosomal recessive enzyme deficiencies result in the deficiency of one or more of the mineralocorticoid, glucocorticoid and/or androgen hormones and the patients present with diverse clinical and laboratory abnormalities depending on the deficient hormone(s) [3].

Although, in more than 95% of the cases, the genetic anomaly is caused by the *CYP21A2* gene encoding the 21α-hydroxylase enzyme, other enzyme deficiencies may also be seen in different ethnic populations [4, 5]. 11β hydroxylase deficiency (11βOHD), which is caused by the mutation in the *CYP11B1* gene, is the second most common (5-8%) form of the disease [6]. Inactivating mutations in this gene result in impaired hydroxylation of the intermediate hormones, namely 11-deoxycortisol and deoxycorticosterone, which eventually leads to the deficiencies of cortisol and corticosterone, respectively [7]. There are two different 11β-hydroxylase isoenzymes (P450c11 and P450c11Aldo), and they share high homology in humans. These two enzymes are highly related, and they have a role in glucocorticoid and mineralocorticoid synthesis, respectively [8]. As in other enzyme deficiencies, patients with 11βOHD also have a classic (residual enzyme activity < 1-2%) and non-classic (residual enzyme activity is 20-50%) form of the disease. In accordance with the severity of enzyme deficiency, patients with 11βOHD may exhibit various degrees of cortisol deficiency, increased adrenal androgens and increased deoxycorticosterone, leading to hypertension [6].

The *CYP11B1* gene encoding 11β-hydroxylase (OMIM #202010) is located on chromosome 8q24.3 with nine exons. More than 100 different mutations in this gene, including splice-site, nonsense/missense, insertions, deletions, and complex rearrangements, have been reported so far and have been recorded in the Human Gene Mutation Database (www.hgmd.cf.ac.uk). Exons 2, 6, 7 and 8 are generally clustered mutation sites among the coding regions [3, 6, 9]. Here, we describe a previously unidentified mutation in a female patient with 11βOH deficient congenital adrenal hyperplasia.

## 2 CASE PRESENTATION

A 35-year-old woman was admitted to the Endocrinology Department of Erciyes University Medical School with an adrenal mass and uncontrolled hypertension. The patient's medical history revealed two genital reconstruction surgery in childhood (at the age of 3 and 15) and high blood pressure, which was first detected at the age of 12. In addition, she was given glucocorticoids in childhood. However, she did not use it regularly afterward and did not use it for the last 5 years. She had regular menses and did not describe any severe complaints in terms of hirsutism. There was consanguinity between her parents. At physical examination, there were hypertension, clitoromegaly, deep voice and mild hirsutism (Ferriman-Gallwey score 10). She took no medication except nifedipine, 60 mg/day, for the treatment of hypertension.

On laboratory evaluation, the patient had hypokalemia (2.7 mmol/L), high serum adrenocorticotropic hormone (ACTH) (279 pg/mL), 11-deoxycortisol (140 ng/ml), total testosterone (236 ng/dL), and androstenedione (> 10 ng/dL) levels. Although the clinical picture was not compatible, in order to exclude pheochromocytoma as a differential diagnosis of adrenal mass, 24-hour urinary free metanephrine and normetanephrine levels were measured, and their levels were 44 μg and 141 μg, respectively (normal ranges: 44-261 μg/24h and 111-419 μg /24h). Some other details of biochemical evaluation are given in Table **1**. Classic 11βOHD was diagnosed with her medical history, physical examination and laboratory evaluation.

**Fig. (1):**
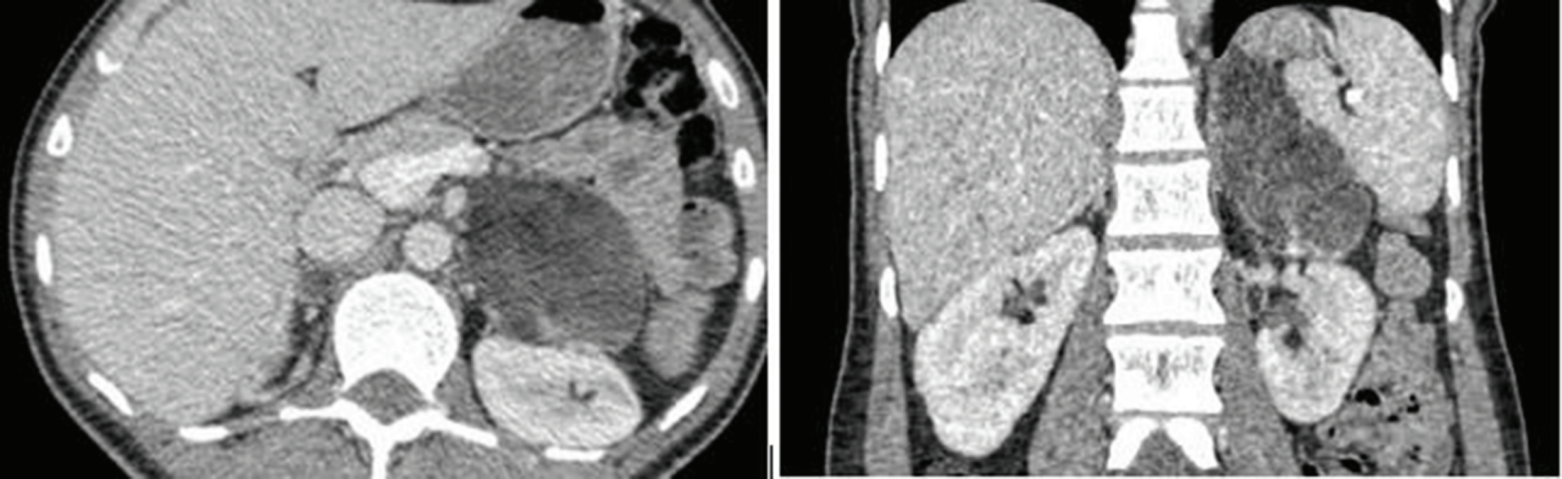
Computed tomography images of the left adrenal mass (74 x 55 mm in size) in axial (**A**) and coronal (**B**) views.

Adrenal computed tomography showed diffuse-moderate thickening and hypodense nodular appearance in the right adrenal gland, while a hypodense nodular mass, 74x55 mm in size, was detected in the left adrenal gland (Fig. **1**). Due to the presence of macroscopic fat densities in the mass, myelolipoma was considered as a preliminary diagnosis of the adrenal mass. Laparoscopic adrenalectomy was performed, and the final diagnosis was myelolipoma, as expected. The patient is currently in her postoperative second year and has been receiving glucocorticoid replacement therapy (Hydrocortisone 15 mg/day). The patient does not have any complaints, and her blood pressure is regulated with glucocorticoid replacement and a single anti-hypertensive (Lercanidipine 20 mg/day). In terms of hormonal and biochemical aspects, serum potassium, 11-deoxycortisol, ACTH and androstenedione levels were within the normal range, and total testosterone was unmeasurably low (Table **1**).

## 3 GENETIC ANALYSIS

**Fig. (2):**
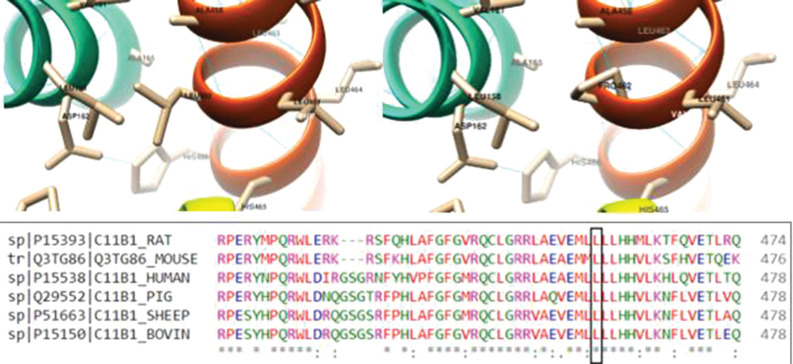
3D visualization of L462P substitution on protein structure. The figure depicts that residue 462 of the CYP11B1 protein is highly conserved among different species.

DNA isolation from the peripheral blood sample was performed using the MagNAPure LC DNA isolation kit (Roche Diagnostic GmbH, Mannheim, Germany) and the MagNAPure LC 2.0 (Roche Diagnostic Ltd. Rotkreuz, Switzerland) device according to the manufacturer's instructions. All protein-coding exons and exon-intron junction regions of the *CYP11B1* gene were sequenced using the Sanger method. The sample was run using the 3500 Genetic Analyzer instrument, and SeqScape Software v3.0 was used for data analysis. Changes were evaluated using the criteria of the American College of Medical Genetics and Genomics and the Association for Molecular Pathology 2015 guidelines [10]. Genetic analysis was performed using Sanger sequencing for the *CYP11B1* gene, revealing a novel homozygous missense mutation (c.1385T > C L462P variant (NM_000497.3). In addition, we used the USCF Chimera v1.15 software to conduct a three-dimensional (3D) modeling analysis to examine the structural level effect of the predicted deleterious change of the new variant [11]. A template of the CYP11B1 protein was taken from Alphafold v2.0 for this application (Fig. **2**) [12].

## 4 DISCUSSION

In this case report, a case of 35 years old Turkish woman characterized by the classical form of CAH due to 11βOHD is presented. Classical and nonclassical form of the disease is relatively rare in comparison to CAH due to 21OHD [13, 14]. To date, more than 100 mutations have been reported on this gene, and our patient exhibited a novel missense mutation, leading to the classical form of the disease.

A female patient with the nonclassical form of 11βOHD presents with signs of mild/moderate hyperandrogenism (oligomenorrhoea/amenorrhea, acne, hirsutism), mimicking polycystic ovary syndrome. Depending on the deficient enzyme, apart from apparently high basal intermediate hormone levels, the ACTH stimulation test has been used for the diagnosis of congenital adrenal hyperplasia [6,15]. Unfortunately, in contrast to 21OHD, there are no clear-cut hormone levels for diagnosing 11βOHD, particularly in the nonclassical phenotype of the disease. Basal or ACTH-stimulated 11-deoxycortisol levels three times above the 95^th^ percentile for the normal population have been suggested for the diagnosis (12 nmol/L), as previously shown [16]. Symptoms related to the classic form of 11βOHD are almost always seen from birth, and the clinical picture is very severe, particularly in females. As a result of the shift of steroid synthesis towards androgens, virilization in the external genitalia in girls and an enlarged penis for age are observed in boys. Testicular adrenal rest tumors may also be seen in the long term when male patients are left untreated or non-adherent to treatment [6, 17]. In addition to the findings related to hyperandrogenism, hypokalemia and hypertension are also common in patients with the classic form of 11βOHD. In fact, these two conditions are critical in differentiating from 21OHD during the differential diagnosis. Depending on the severity of the mutation, elevated deoxycorticosterone levels mimic aldosteronism, thereby increasing blood pressure and causing hypokalemia. However, it is important to note that treatment with glucocorticoids may precipitate actual mineralocorticoid deficiency due to the suppression of deoxycorticosterone [18].

In this case report, we detected a novel homozygous missense mutation (c.1385T > C (L462P) in exon 8 of the *CYP11B1* gene by using Sanger sequencing. Although exon 8 is one of the clustered mutation sites, this variant has never been reported before in the gnomAD exomes, 1000 Genomes and ClinVar databases. Moreover, *in silico* tools, DANN, FATHMM-MKL, Mutation Assessor, Mutation Taster, and SIFT, predict pathogenicity, while only PrimateAI shows as benign. The REVEL score is a tool used to predict the pathogenicity of missense variants and is moderately pathogenic (0.8) for this new variant. Furthermore, Fig. (**2**) shows that residue 462 of the CYP11B1 protein is highly conserved across species. Therefore, we interpreted the c.1385T > C L462P variant as of uncertain clinical significance. More than 100 mutations causing 11βOHD have been identified in the CYP11B1 gene, consisting of 9 exons encoding 503 amino acids [6, 19]. These mutations are not of a single type but vary widely and include missense/nonsense, splicing, deletions, insertions and complex rearrangements. Unlike clinical and genetic studies on 21 OHD, 11βOHD is rarely investigated due to its rarity in most communities.

Congenital adrenal hyperplasia due to 11βOHD is more common in some communities, including Turkey, because of the higher consanguineous marriages [20, 21]. To our knowledge, this mutation has not been reported previously, and since there was no known mutation in this patient compatible with classical features of 11βOHD, we think that this novel mutation is likely pathogenic. It is remarkable that although she had genital reconstructive surgery indicative of severe hyperandrogenism, she has neither suffered from severe hirsutism nor severe menstrual disturbances. It has been shown that there is no clear correlation between a specific mutation and clinical presentation. The same mutation may present diverse signs and symptoms related to androgen and mineralocorticoid activities [8, 22].

Although the patient has a classic form of the disease, unfortunately, she was not aware of her disease and came to the clinic due to an adrenal mass lesion. We should emphasize that the imaging finding of this patient is not directly related to her disorder. Adrenal myelolipomas are benign lipomatous tumors that comprise 3-6% of all adrenal masses. Although bilateral hyperplastic adrenal glands are commonly seen in patients with classical CAH, those patients may exhibit a high prevalence of association with adrenal myelolipomas than any other patient group [23].

## CONCLUSION

We described a novel missense mutation in the CYP11B1 gene, leading to the classical form of 11βOHD. This case report expanded the mutational spectrum of CYP11B1 and may help to accurately diagnose 11βOHD.

## Figures and Tables

**Table 1:** Laboratory findings of the patient on her first admission and post-operative second year.

-	**Normal Range**	**Patient’s Level**	
-	-	**First Admission**	**Post-operative 2^nd^ Year**
Potassium (mmol/L)	3.5-5.1	2.7	4
Sodium (mmol/L)	136-145	142	140
Cortisol (µg/dL)	6,2-18	4.8	0.4
ACTH (pg/mL)	0-46	279	18
11-deoxycortisol (ng/ml)	0-0,4	140	0.3
17-OH-progesterone (ng/ml)	0.2-1: follicular phase 0.2-3: luteal phase	8.31	2.7
Androstenedione (ng/dl)	0,3-3,3	>10	1.2
DHEA-S (μg/dl)	61-337	237	68
Total Testosterone (ng/dl)	(6-82)	236	< 2.5
FSH (mIU/mL)	1.9-76.3	6.8	-
LH (mIU/mL)	1.5-33.4	3.8	-
Estradiol (pg/ml)	12.4-398	37.7	-

## Data Availability

The data are available from the corresponding author upon reasonable request.

## LIST OF ABBREVIATIONS

CAH: Congenital Adrenal Hyperplasia
11βOHD: 11β Hydroxylase Deficiency
ACTH: Adrenocorticotropic Hormone
